# *catena*-Poly[dipropyl­ammonium [[bis­(benzotriazolato-κ*N* ^1^)zinc(II)]-μ-benzotriazolato-κ^2^ *N* ^1^:*N* ^3^]]

**DOI:** 10.1107/S160053680902563X

**Published:** 2009-07-08

**Authors:** Liping Xu

**Affiliations:** a College of Science and Information, Qingdao Agricultural University, No. 700 Changcheng Road, Chengyang, Qingdao 266109, People’s Republic of China

## Abstract

In the title compound, {(C_6_H_16_N)[Zn(C_6_H_4_N_3_)_3_]}_*n*_, the Zn^II^ atom has a distorted tetra­hedral geometry defined by four N atoms from four benzotriazolate (BTA) ligands. The compound is composed of extended polymeric chains in which two BTA N atoms bridge [Zn(BTA)_2_] fragments along [001]. Cations and anions are linked by N—H⋯N hydrogen-bond inter­actions along [010].

## Related literature

For background information on the design and synthesis of supra­molecular complexes: see Yaghi *et al.* (1998 ▶); Shao *et al.* (2008 ▶).
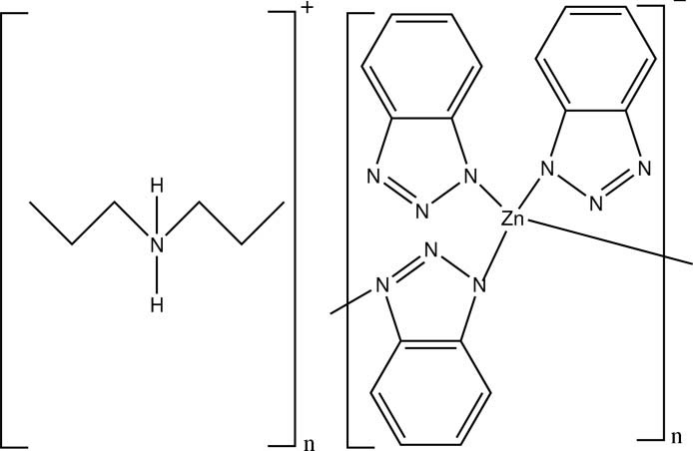


## Experimental

### 

#### Crystal data

(C_6_H_16_N)[Zn(C_6_H_4_N_3_)_3_]
*M* *_r_* = 521.93
Monoclinic, 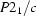

*a* = 11.9439 (15) Å
*b* = 9.8058 (13) Å
*c* = 21.585 (3) Å
β = 104.212 (2)°
*V* = 2450.7 (5) Å^3^
*Z* = 4
Mo *K*α radiation
μ = 1.04 mm^−1^
*T* = 293 K
0.35 × 0.20 × 0.06 mm

#### Data collection

Bruker SMART APEX CCD area-detector diffractometer
Absorption correction: multi-scan (*SADABS*; Sheldrick, 1996 ▶) *T*_min_ = 0.286, *T*_max_ = 0.322 (expected range = 0.835–0.940)
11639 measured reflections
4107 independent reflections
2645 reflections with *I* > 2σ(*I*)
*R*_int_ = 0.075

#### Refinement

*R*[*F* ^2^ > 2σ(*F* ^2^)] = 0.042
*wR*(*F* ^2^) = 0.095
*S* = 0.96
4107 reflections
316 parameters
H-atom parameters constrained
Δρ_max_ = 0.53 e Å^−3^
Δρ_min_ = −0.54 e Å^−3^

### 

Data collection: *SMART* (Bruker, 1999 ▶); cell refinement: *SAINT* (Bruker, 1999 ▶); data reduction: *SAINT*; program(s) used to solve structure: *SHELXS97* (Sheldrick, 2008 ▶); program(s) used to refine structure: *SHELXL97* (Sheldrick, 2008 ▶); molecular graphics: *SHELXTL-Plus* (Sheldrick, 2008 ▶); software used to prepare material for publication: *SHELXL97*.

## Supplementary Material

Crystal structure: contains datablocks I, global. DOI: 10.1107/S160053680902563X/bx2217sup1.cif

Structure factors: contains datablocks I. DOI: 10.1107/S160053680902563X/bx2217Isup2.hkl

Additional supplementary materials:  crystallographic information; 3D view; checkCIF report

## Figures and Tables

**Table:** 

N1—Zn1^i^	2.000 (3)
N3—Zn1	1.990 (3)
N6—Zn1	1.982 (3)
Zn1—N1^ii^	2.000 (3)

**Table:** 

N6—Zn1—N1^ii^	106.15 (12)
N9—Zn1—N1^ii^	111.79 (12)
N3—Zn1—N1^ii^	113.61 (12)

Symmetry codes: (i) 
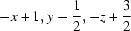
; (ii) 
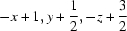
.

**Table 2:** Hydrogen-bond geometry (Å, °)

*D*—H⋯*A*	*D*—H	H⋯*A*	*D*⋯*A*	*D*—H⋯*A*
N10—H27⋯N7	0.90	1.96	2.824 (4)	160
N10—H27⋯N8	0.90	2.42	3.052 (4)	127
N10—H26⋯N4^iii^	0.90	1.93	2.821 (4)	171

Symmetry code: (iii) 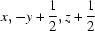.

**Fig. 1.**
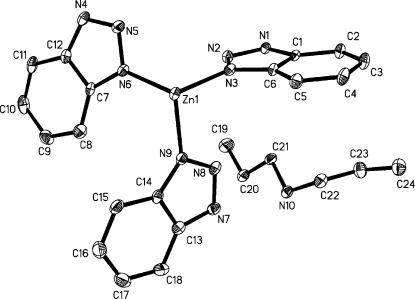
The asymmetric unit of I. Displacement ellipsoids are drawn at the 30% probability level and all H atoms have been omitted for clarity.

## supplementary crystallographic information

### Comment

In the research of supramolecular chemistry, a great interest has recently been focused on the crystal engineering of coordination frameworks due to their intriguing architectures, new topologies, intertwining phenomena and potential applications in microelectronics, nonlinear optics, ion exchange, molecular selection, molecular separation and recognition (Yaghi *et al.*, 1998). Shao *et al.* report the first example of chiral [Zn(BTA)_2_] with a bikitaite (BIK) zeolitic topology, was successfully isolated under hydrothermal conditions (Shao *et al.*, 2008). Here, we report the crystal and molecular structure of {[DPAH][Zn(BTA)_3_]}_n_ , (I), Fig. 1 (DPAH=dipropylammonium, BTA = benzotriazolate).The Zn atom has a distorted tetrahedral geometry, defined by four N atoms from four BTA ligands. The BTA behaves as a monodentate ligand.The material is composed of one-dimensional extended polymeric chains in which two N atoms from benzotriazolate anion bridges fragment of Zn(BTA)_2 _in [0 0 1] direction. The cations and anions are linked by N-H···N hydrogen bond interaction in the [0 1 0 ] direction.

### Experimental

A mixture of Zn(NO_3_)_2_. 6H_2_O (0.149 g, 0.5 mmol), BTAH (0.119 g, 1.0 mmol) in 3.0 ml DPA solution was stirred for 30 min in a 17.0 ml Teflon-lined stainless steel. The vessel was sealed and heated at 190 °C for one week. The orange block crystals were isolated by washing with ethanol and water.

### Refinement

Hydrogen atoms were placed at calculated positions (0.90–0.97 Å) and were treated as riding on their parent atoms, with U(H) set to 1.2*U*_eq_(C/N).

### Crystal data

**Table:**

(C_6_H_16_N)[Zn(C_6_H_4_N_3_)_3_]	*F*(000) = 1088
*M**_r_* = 521.93	*D*_x_ = 1.415 Mg m^−^^3^
Monoclinic, *P*2_1_/*c*	Mo *K*α radiation, λ = 0.71073 Å
Hall symbol: -P 2ybc	Cell parameters from 12055 reflections
*a* = 11.9439 (15) Å	θ = 2.0–24.6°
*b* = 9.8058 (13) Å	µ = 1.04 mm^−^^1^
*c* = 21.585 (3) Å	*T* = 293 K
β = 104.212 (2)°	Plate, white
*V* = 2450.7 (5) Å^3^	0.35 × 0.2 × 0.06 mm
*Z* = 4	

### Data collection

**Table:**

Bruker SMART APEX CCD area-detector diffractometer	4107 independent reflections
Radiation source: fine-focus sealed tube	2645 reflections with *I* > 2σ(*I*)
graphite	*R*_int_ = 0.075
ω scans	θ_max_ = 24.6°, θ_min_ = 2.0°
Absorption correction: multi-scan (*SADABS*; Sheldrick, 1996)	*h* = −13→13
*T*_min_ = 0.286, *T*_max_ = 0.322	*k* = −11→11
11639 measured reflections	*l* = −14→25

### Refinement

**Table:**

Refinement on *F*^2^	Primary atom site location: structure-invariant direct methods
Least-squares matrix: full	Secondary atom site location: difference Fourier map
*R*[*F*^2^ > 2σ(*F*^2^)] = 0.042	Hydrogen site location: inferred from neighbouring sites
*wR*(*F*^2^) = 0.095	H-atom parameters constrained
*S* = 0.96	*w* = 1/[σ^2^(*F*_o_^2^) + (0.0376*P*)^2^] where *P* = (*F*_o_^2^ + 2*F*_c_^2^)/3
4107 reflections	(Δ/σ)_max_ = 0.001
316 parameters	Δρ_max_ = 0.53 e Å^−^^3^
0 restraints	Δρ_min_ = −0.53 e Å^−^^3^

### Special details

**Table:**

Geometry. All e.s.d.'s (except the e.s.d. in the dihedral angle between two l.s. planes) are estimated using the full covariance matrix. The cell e.s.d.'s are taken into account individually in the estimation of e.s.d.'s in distances, angles and torsion angles; correlations between e.s.d.'s in cell parameters are only used when they are defined by crystal symmetry. An approximate (isotropic) treatment of cell e.s.d.'s is used for estimating e.s.d.'s involving l.s. planes.
Refinement. Refinement of *F*^2^ against ALL reflections. The weighted *R*-factor *wR* and goodness of fit *S* are based on *F*^2^, conventional *R*-factors *R* are based on *F*, with *F* set to zero for negative *F*^2^. The threshold expression of *F*^2^ > σ(*F*^2^) is used only for calculating *R*-factors(gt) *etc*. and is not relevant to the choice of reflections for refinement. *R*-factors based on *F*^2^ are statistically about twice as large as those based on *F*, and *R*- factors based on ALL data will be even larger.

### Fractional atomic coordinates and isotropic or equivalent isotropic displacement parameters (Å^2^)

**Table:**

	*x*	*y*	*z*	*U*_iso_*/*U*_eq_	
C1	0.6479 (3)	0.3188 (4)	0.83315 (16)	0.0186 (9)	
C2	0.7447 (3)	0.3069 (4)	0.88465 (17)	0.0261 (10)	
H2	0.7856	0.2256	0.8936	0.031*	
C3	0.7760 (4)	0.4204 (4)	0.92092 (18)	0.0299 (10)	
H3	0.8402	0.4166	0.9554	0.036*	
C4	0.7145 (3)	0.5429 (4)	0.90794 (17)	0.0315 (10)	
H4	0.7387	0.6179	0.9342	0.038*	
C5	0.6201 (3)	0.5554 (4)	0.85786 (17)	0.0279 (10)	
H5	0.5793	0.6368	0.8493	0.033*	
C6	0.5877 (3)	0.4396 (4)	0.81994 (17)	0.0199 (9)	
C7	0.1821 (3)	0.4217 (3)	0.60887 (17)	0.0204 (9)	
C8	0.0800 (4)	0.4131 (4)	0.6293 (2)	0.0325 (10)	
H8	0.0773	0.4388	0.6704	0.039*	
C9	−0.0156 (4)	0.3656 (4)	0.5864 (2)	0.0380 (11)	
H9	−0.0846	0.3580	0.5988	0.046*	
C10	−0.0127 (4)	0.3280 (4)	0.5245 (2)	0.0388 (11)	
H10	−0.0800	0.2968	0.4966	0.047*	
C11	0.0852 (4)	0.3356 (4)	0.50407 (19)	0.0333 (11)	
H11	0.0866	0.3100	0.4628	0.040*	
C12	0.1840 (3)	0.3832 (4)	0.54704 (17)	0.0242 (9)	
C13	0.1248 (3)	0.4877 (4)	0.83554 (17)	0.0255 (10)	
C14	0.1554 (3)	0.5604 (4)	0.78660 (17)	0.0244 (10)	
C15	0.0800 (4)	0.6553 (4)	0.75005 (18)	0.0314 (10)	
H15	0.1003	0.7054	0.7179	0.038*	
C16	−0.0249 (4)	0.6710 (4)	0.7637 (2)	0.0400 (12)	
H16	−0.0774	0.7330	0.7402	0.048*	
C17	−0.0556 (4)	0.5959 (5)	0.8124 (2)	0.0418 (12)	
H17	−0.1284	0.6087	0.8198	0.050*	
C18	0.0170 (3)	0.5059 (4)	0.84886 (19)	0.0346 (11)	
H18	−0.0037	0.4579	0.8815	0.042*	
C19	0.2867 (4)	−0.0562 (4)	0.8168 (2)	0.0502 (13)	
H20	0.2170	−0.1089	0.8047	0.075*	
H21	0.3514	−0.1161	0.8313	0.075*	
H19	0.2968	−0.0051	0.7806	0.075*	
C20	0.2788 (3)	0.0409 (4)	0.86994 (18)	0.0338 (10)	
H23	0.2128	0.1006	0.8552	0.041*	
H22	0.2664	−0.0109	0.9060	0.041*	
C21	0.3860 (3)	0.1259 (4)	0.89145 (18)	0.0282 (10)	
H24	0.4518	0.0673	0.9085	0.034*	
H25	0.4006	0.1756	0.8554	0.034*	
C22	0.4728 (3)	0.3076 (4)	0.97182 (18)	0.0310 (10)	
H28	0.4487	0.3792	0.9967	0.037*	
H29	0.5027	0.3506	0.9388	0.037*	
C23	0.5670 (4)	0.2259 (4)	1.0144 (2)	0.0379 (11)	
H31	0.6029	0.1676	0.9885	0.045*	
H30	0.5343	0.1681	1.0419	0.045*	
C24	0.6569 (4)	0.3179 (4)	1.0548 (2)	0.0414 (12)	
H33	0.7161	0.2636	1.0818	0.062*	
H32	0.6215	0.3751	1.0807	0.062*	
H34	0.6905	0.3738	1.0275	0.062*	
N1	0.5952 (3)	0.2264 (3)	0.78762 (14)	0.0210 (7)	
N2	0.5060 (3)	0.2896 (3)	0.74886 (15)	0.0219 (7)	
N3	0.5003 (3)	0.4185 (3)	0.76708 (14)	0.0211 (8)	
N4	0.2938 (3)	0.4007 (3)	0.54137 (14)	0.0290 (8)	
N5	0.3569 (3)	0.4464 (3)	0.59662 (15)	0.0283 (8)	
N6	0.2912 (2)	0.4601 (3)	0.63867 (13)	0.0214 (7)	
N7	0.2144 (3)	0.4034 (3)	0.86208 (15)	0.0284 (8)	
N8	0.2947 (3)	0.4209 (3)	0.82976 (14)	0.0279 (8)	
N9	0.2615 (3)	0.5154 (3)	0.78374 (14)	0.0229 (7)	
N10	0.3708 (3)	0.2231 (3)	0.94134 (14)	0.0259 (8)	
H26	0.3495	0.1755	0.9722	0.031*	
H27	0.3122	0.2795	0.9238	0.031*	
Zn1	0.36481 (4)	0.53198 (4)	0.72486 (2)	0.02055 (14)	

### Atomic displacement parameters (Å^2^)

**Table:**

	*U*^11^	*U*^22^	*U*^33^	*U*^12^	*U*^13^	*U*^23^
C1	0.023 (2)	0.019 (2)	0.014 (2)	−0.0020 (18)	0.0035 (17)	0.0010 (17)
C2	0.032 (2)	0.023 (2)	0.020 (2)	0.005 (2)	−0.0017 (19)	−0.0009 (18)
C3	0.035 (3)	0.028 (2)	0.021 (2)	0.004 (2)	−0.004 (2)	0.0037 (19)
C4	0.041 (3)	0.028 (2)	0.020 (2)	−0.006 (2)	−0.002 (2)	−0.005 (2)
C5	0.032 (2)	0.025 (3)	0.025 (2)	0.001 (2)	0.0039 (19)	0.0051 (19)
C6	0.024 (2)	0.019 (2)	0.014 (2)	−0.0004 (18)	0.0005 (17)	0.0008 (16)
C7	0.026 (2)	0.017 (2)	0.015 (2)	−0.0019 (18)	−0.0014 (18)	0.0017 (16)
C8	0.036 (3)	0.032 (3)	0.028 (2)	−0.004 (2)	0.005 (2)	−0.0056 (19)
C9	0.023 (3)	0.045 (3)	0.044 (3)	−0.006 (2)	0.003 (2)	−0.004 (2)
C10	0.035 (3)	0.034 (3)	0.035 (3)	−0.006 (2)	−0.015 (2)	−0.009 (2)
C11	0.043 (3)	0.030 (3)	0.020 (2)	−0.001 (2)	−0.006 (2)	−0.0049 (19)
C12	0.030 (3)	0.019 (2)	0.020 (2)	−0.0021 (19)	−0.0008 (19)	0.0009 (18)
C13	0.031 (2)	0.021 (2)	0.024 (2)	−0.0039 (19)	0.0065 (19)	−0.0049 (18)
C14	0.029 (2)	0.024 (2)	0.018 (2)	−0.0020 (19)	0.0011 (19)	−0.0032 (17)
C15	0.039 (3)	0.035 (3)	0.019 (2)	0.002 (2)	0.006 (2)	−0.0032 (19)
C16	0.038 (3)	0.042 (3)	0.035 (3)	0.008 (2)	0.001 (2)	−0.003 (2)
C17	0.032 (3)	0.047 (3)	0.047 (3)	0.004 (2)	0.011 (2)	−0.010 (2)
C18	0.036 (3)	0.040 (3)	0.033 (3)	0.000 (2)	0.016 (2)	−0.002 (2)
C19	0.054 (3)	0.056 (3)	0.042 (3)	0.000 (3)	0.015 (3)	−0.010 (3)
C20	0.036 (3)	0.041 (3)	0.024 (2)	−0.002 (2)	0.007 (2)	−0.007 (2)
C21	0.034 (3)	0.031 (2)	0.022 (2)	0.006 (2)	0.010 (2)	0.0035 (19)
C22	0.037 (3)	0.028 (2)	0.028 (2)	−0.005 (2)	0.007 (2)	−0.001 (2)
C23	0.036 (3)	0.035 (3)	0.039 (3)	−0.004 (2)	0.002 (2)	−0.002 (2)
C24	0.040 (3)	0.044 (3)	0.037 (3)	−0.005 (2)	0.002 (2)	−0.001 (2)
N1	0.0237 (19)	0.0212 (18)	0.0172 (18)	0.0012 (15)	0.0033 (15)	0.0035 (14)
N2	0.0254 (18)	0.0170 (17)	0.0217 (17)	0.0012 (14)	0.0028 (14)	0.0005 (14)
N3	0.028 (2)	0.0172 (18)	0.0177 (18)	−0.0031 (15)	0.0058 (15)	−0.0024 (14)
N4	0.038 (2)	0.032 (2)	0.0156 (18)	−0.0014 (17)	0.0039 (17)	−0.0031 (15)
N5	0.030 (2)	0.033 (2)	0.0221 (18)	−0.0009 (17)	0.0077 (16)	−0.0014 (16)
N6	0.0252 (18)	0.0213 (17)	0.0174 (17)	−0.0031 (16)	0.0047 (14)	−0.0004 (15)
N7	0.035 (2)	0.029 (2)	0.0232 (19)	0.0002 (17)	0.0106 (17)	0.0042 (16)
N8	0.034 (2)	0.030 (2)	0.0195 (18)	0.0002 (16)	0.0051 (16)	0.0053 (15)
N9	0.0264 (19)	0.0230 (18)	0.0184 (17)	0.0024 (16)	0.0038 (14)	−0.0006 (15)
N10	0.031 (2)	0.0256 (19)	0.0213 (19)	0.0012 (16)	0.0062 (16)	0.0054 (15)
Zn1	0.0255 (3)	0.0186 (2)	0.0158 (2)	−0.0003 (2)	0.00162 (18)	−0.0001 (2)

### Geometric parameters (Å, °)

**Table:**

C1—N1	1.370 (4)	C19—H20	0.9600
C1—C6	1.379 (5)	C19—H21	0.9600
C1—C2	1.398 (5)	C19—H19	0.9600
C2—C3	1.359 (5)	C20—C21	1.503 (5)
C2—H2	0.9300	C20—H23	0.9700
C3—C4	1.400 (5)	C20—H22	0.9700
C3—H3	0.9300	C21—N10	1.482 (4)
C4—C5	1.362 (5)	C21—H24	0.9700
C4—H4	0.9300	C21—H25	0.9700
C5—C6	1.398 (5)	C22—N10	1.486 (4)
C5—H5	0.9300	C22—C23	1.498 (5)
C6—N3	1.360 (4)	C22—H28	0.9700
C7—N6	1.357 (4)	C22—H29	0.9700
C7—C12	1.392 (5)	C23—C24	1.505 (5)
C7—C8	1.398 (5)	C23—H31	0.9700
C8—C9	1.362 (5)	C23—H30	0.9700
C8—H8	0.9300	C24—H33	0.9600
C9—C10	1.396 (5)	C24—H32	0.9600
C9—H9	0.9300	C24—H34	0.9600
C10—C11	1.350 (6)	N1—N2	1.334 (4)
C10—H10	0.9300	N1—Zn1^i^	2.000 (3)
C11—C12	1.390 (5)	N2—N3	1.331 (4)
C11—H11	0.9300	N3—Zn1	1.990 (3)
C12—N4	1.357 (5)	N4—N5	1.323 (4)
C13—N7	1.362 (5)	N5—N6	1.345 (4)
C13—N7	1.362 (5)	N6—Zn1	1.982 (3)
C13—C14	1.396 (5)	N7—N7	0.000 (7)
C13—C18	1.398 (5)	N7—N8	1.327 (4)
C14—N9	1.358 (4)	N7—N8	1.327 (4)
C14—C15	1.397 (5)	N8—N8	0.000 (9)
C15—C16	1.364 (5)	N8—N7	1.327 (4)
C15—H15	0.9300	N8—N9	1.345 (4)
C16—C17	1.404 (6)	N9—N8	1.345 (4)
C16—H16	0.9300	N9—Zn1	1.983 (3)
C17—C18	1.347 (5)	N10—H26	0.9000
C17—H17	0.9300	N10—H27	0.9000
C18—H18	0.9300	Zn1—N1^ii^	2.000 (3)
C19—C20	1.512 (5)		
			
N1—C1—C6	107.2 (3)	H23—C20—H22	107.9
N1—C1—C2	131.3 (3)	N10—C21—C20	109.5 (3)
C6—C1—C2	121.6 (4)	N10—C21—H24	109.8
C3—C2—C1	116.5 (4)	C20—C21—H24	109.8
C3—C2—H2	121.7	N10—C21—H25	109.8
C1—C2—H2	121.7	C20—C21—H25	109.8
C2—C3—C4	122.1 (4)	H24—C21—H25	108.2
C2—C3—H3	119.0	N10—C22—C23	112.6 (3)
C4—C3—H3	119.0	N10—C22—H28	109.1
C5—C4—C3	121.8 (4)	C23—C22—H28	109.1
C5—C4—H4	119.1	N10—C22—H29	109.1
C3—C4—H4	119.1	C23—C22—H29	109.1
C4—C5—C6	116.6 (4)	H28—C22—H29	107.8
C4—C5—H5	121.7	C22—C23—C24	110.9 (4)
C6—C5—H5	121.7	C22—C23—H31	109.5
N3—C6—C1	107.3 (3)	C24—C23—H31	109.5
N3—C6—C5	131.3 (3)	C22—C23—H30	109.5
C1—C6—C5	121.4 (4)	C24—C23—H30	109.5
N6—C7—C12	106.9 (3)	H31—C23—H30	108.1
N6—C7—C8	132.7 (3)	C23—C24—H33	109.5
C12—C7—C8	120.4 (4)	C23—C24—H32	109.5
C9—C8—C7	117.2 (4)	H33—C24—H32	109.5
C9—C8—H8	121.4	C23—C24—H34	109.5
C7—C8—H8	121.4	H33—C24—H34	109.5
C8—C9—C10	121.8 (4)	H32—C24—H34	109.5
C8—C9—H9	119.1	N2—N1—C1	107.3 (3)
C10—C9—H9	119.1	N2—N1—Zn1^i^	122.6 (2)
C11—C10—C9	121.7 (4)	C1—N1—Zn1^i^	130.1 (2)
C11—C10—H10	119.2	N3—N2—N1	110.3 (3)
C9—C10—H10	119.2	N2—N3—C6	107.9 (3)
C10—C11—C12	117.6 (4)	N2—N3—Zn1	119.4 (2)
C10—C11—H11	121.2	C6—N3—Zn1	132.0 (2)
C12—C11—H11	121.2	N5—N4—C12	108.1 (3)
N4—C12—C11	131.5 (4)	N4—N5—N6	110.2 (3)
N4—C12—C7	107.2 (3)	N5—N6—C7	107.7 (3)
C11—C12—C7	121.3 (4)	N5—N6—Zn1	118.1 (2)
N7—C13—N7	0.0 (3)	C7—N6—Zn1	134.2 (2)
N7—C13—C14	107.7 (3)	N7—N7—N8	0(10)
N7—C13—C14	107.7 (3)	N7—N7—N8	0(10)
N7—C13—C18	131.2 (4)	N8—N7—N8	0.00 (19)
N7—C13—C18	131.2 (4)	N7—N7—C13	0(10)
C14—C13—C18	121.1 (4)	N8—N7—C13	107.1 (3)
N9—C14—C13	106.8 (3)	N8—N7—C13	107.1 (3)
N9—C14—C15	132.2 (4)	N8—N8—N7	0(10)
C13—C14—C15	121.0 (4)	N8—N8—N7	0(10)
C16—C15—C14	116.9 (4)	N7—N8—N7	0.0 (3)
C16—C15—H15	121.5	N8—N8—N9	0(10)
C14—C15—H15	121.5	N7—N8—N9	110.9 (3)
C15—C16—C17	121.7 (4)	N7—N8—N9	110.9 (3)
C15—C16—H16	119.2	N8—N9—N8	0.0 (3)
C17—C16—H16	119.2	N8—N9—C14	107.4 (3)
C18—C17—C16	122.1 (4)	N8—N9—C14	107.4 (3)
C18—C17—H17	118.9	N8—N9—Zn1	114.4 (2)
C16—C17—H17	118.9	N8—N9—Zn1	114.4 (2)
C17—C18—C13	117.2 (4)	C14—N9—Zn1	137.5 (3)
C17—C18—H18	121.4	C21—N10—C22	116.5 (3)
C13—C18—H18	121.4	C21—N10—H26	108.2
C20—C19—H20	109.5	C22—N10—H26	108.2
C20—C19—H21	109.5	C21—N10—H27	108.2
H20—C19—H21	109.5	C22—N10—H27	108.2
C20—C19—H19	109.5	H26—N10—H27	107.3
H20—C19—H19	109.5	N6—Zn1—N9	111.71 (12)
H21—C19—H19	109.5	N6—Zn1—N3	110.77 (12)
C21—C20—C19	112.1 (3)	N9—Zn1—N3	102.96 (12)
C21—C20—H23	109.2	N6—Zn1—N1^ii^	106.15 (12)
C19—C20—H23	109.2	N9—Zn1—N1^ii^	111.79 (12)
C21—C20—H22	109.2	N3—Zn1—N1^ii^	113.61 (12)
C19—C20—H22	109.2		
			
N1—C1—C2—C3	179.1 (4)	C8—C7—N6—N5	178.8 (4)
C6—C1—C2—C3	0.2 (5)	C12—C7—N6—Zn1	178.4 (3)
C1—C2—C3—C4	0.4 (6)	C8—C7—N6—Zn1	−3.1 (6)
C2—C3—C4—C5	−0.4 (6)	C14—C13—N7—N7	0.0 (5)
C3—C4—C5—C6	−0.1 (6)	C18—C13—N7—N7	0.0 (5)
N1—C1—C6—N3	0.0 (4)	N7—C13—N7—N8	0(100)
C2—C1—C6—N3	179.2 (3)	C14—C13—N7—N8	−1.7 (4)
N1—C1—C6—C5	−179.8 (3)	C18—C13—N7—N8	176.8 (4)
C2—C1—C6—C5	−0.6 (6)	N7—C13—N7—N8	0(100)
C4—C5—C6—N3	−179.2 (4)	C14—C13—N7—N8	−1.7 (4)
C4—C5—C6—C1	0.6 (5)	C18—C13—N7—N8	176.8 (4)
N6—C7—C8—C9	−178.1 (4)	N7—N7—N8—N8	0.0
C12—C7—C8—C9	0.3 (6)	C13—N7—N8—N8	0.0 (9)
C7—C8—C9—C10	−0.6 (6)	N8—N7—N8—N7	0(100)
C8—C9—C10—C11	0.6 (7)	C13—N7—N8—N7	0(100)
C9—C10—C11—C12	−0.3 (6)	N7—N7—N8—N9	0.0 (10)
C10—C11—C12—N4	179.0 (4)	N8—N7—N8—N9	0(100)
C10—C11—C12—C7	0.0 (6)	C13—N7—N8—N9	1.1 (4)
N6—C7—C12—N4	−0.5 (4)	N7—N8—N9—N8	0(100)
C8—C7—C12—N4	−179.2 (3)	N7—N8—N9—N8	0(100)
N6—C7—C12—C11	178.8 (3)	N8—N8—N9—C14	0.0 (9)
C8—C7—C12—C11	0.0 (6)	N7—N8—N9—C14	−0.1 (4)
N7—C13—C14—N9	1.7 (4)	N7—N8—N9—C14	−0.1 (4)
N7—C13—C14—N9	1.7 (4)	N8—N8—N9—Zn1	0.0 (9)
C18—C13—C14—N9	−177.0 (3)	N7—N8—N9—Zn1	−172.1 (2)
N7—C13—C14—C15	179.7 (3)	N7—N8—N9—Zn1	−172.1 (2)
N7—C13—C14—C15	179.7 (3)	C13—C14—N9—N8	−1.0 (4)
C18—C13—C14—C15	1.0 (6)	C15—C14—N9—N8	−178.7 (4)
N9—C14—C15—C16	176.2 (4)	C13—C14—N9—N8	−1.0 (4)
C13—C14—C15—C16	−1.2 (6)	C15—C14—N9—N8	−178.7 (4)
C14—C15—C16—C17	0.3 (6)	C13—C14—N9—Zn1	168.3 (3)
C15—C16—C17—C18	0.9 (7)	C15—C14—N9—Zn1	−9.5 (7)
C16—C17—C18—C13	−1.2 (6)	C20—C21—N10—C22	175.1 (3)
N7—C13—C18—C17	−178.1 (4)	C23—C22—N10—C21	−70.4 (4)
N7—C13—C18—C17	−178.1 (4)	N5—N6—Zn1—N9	−170.4 (2)
C14—C13—C18—C17	0.2 (6)	C7—N6—Zn1—N9	11.7 (4)
C19—C20—C21—N10	177.4 (3)	N5—N6—Zn1—N3	−56.3 (3)
N10—C22—C23—C24	−168.0 (3)	C7—N6—Zn1—N3	125.8 (3)
C6—C1—N1—N2	−0.5 (4)	N5—N6—Zn1—N1^ii^	67.5 (3)
C2—C1—N1—N2	−179.5 (4)	C7—N6—Zn1—N1^ii^	−110.4 (3)
C6—C1—N1—Zn1^i^	179.0 (2)	N8—N9—Zn1—N6	109.2 (2)
C2—C1—N1—Zn1^i^	−0.1 (6)	N8—N9—Zn1—N6	109.2 (2)
C1—N1—N2—N3	0.8 (4)	C14—N9—Zn1—N6	−59.6 (4)
Zn1^i^—N1—N2—N3	−178.7 (2)	N8—N9—Zn1—N3	−9.7 (3)
N1—N2—N3—C6	−0.8 (4)	N8—N9—Zn1—N3	−9.7 (3)
N1—N2—N3—Zn1	−172.5 (2)	C14—N9—Zn1—N3	−178.5 (4)
C1—C6—N3—N2	0.5 (4)	N8—N9—Zn1—N1^ii^	−132.0 (2)
C5—C6—N3—N2	−179.7 (4)	N8—N9—Zn1—N1^ii^	−132.0 (2)
C1—C6—N3—Zn1	170.8 (2)	C14—N9—Zn1—N1^ii^	59.2 (4)
C5—C6—N3—Zn1	−9.4 (6)	N2—N3—Zn1—N6	−23.7 (3)
C11—C12—N4—N5	−178.7 (4)	C6—N3—Zn1—N6	166.9 (3)
C7—C12—N4—N5	0.5 (4)	N2—N3—Zn1—N9	95.9 (3)
C12—N4—N5—N6	−0.3 (4)	C6—N3—Zn1—N9	−73.5 (3)
N4—N5—N6—C7	0.0 (4)	N2—N3—Zn1—N1^ii^	−143.0 (2)
N4—N5—N6—Zn1	−178.4 (2)	C6—N3—Zn1—N1^ii^	47.6 (3)
C12—C7—N6—N5	0.3 (4)		

Symmetry codes:  (i) −*x*+1, *y*−1/2, −*z*+3/2; (ii) −*x*+1, *y*+1/2, −*z*+3/2.

### Hydrogen-bond geometry (Å, °)

**Table:**

*D*—H···*A*	*D*—H	H···*A*	*D*···*A*	*D*—H···*A*
N10—H27···N7	0.90	1.96	2.824 (4)	160
N10—H27···N8	0.90	2.42	3.052 (4)	127
N10—H26···N4^iii^	0.90	1.93	2.821 (4)	171

Symmetry codes:  (iii) *x*, −*y*+1/2, *z*+1/2.
